# Actual and Perceived Level of Scientific English across Italian Physiotherapy Courses: A Cross-Sectional Study

**DOI:** 10.3390/healthcare9091135

**Published:** 2021-08-31

**Authors:** Raffaele Cutolo, Simone Battista, Marco Testa

**Affiliations:** 1 School of Medicine and Surgery, University of Verona, 37135 Verona, Italy; raffaele.cutolo@univr.it; 2 Department of Neurosciences, Rehabilitation, Ophthalmology, Genetics, Maternal and Child Health, University of Genova, Campus of Savona, 17100 Savona, Italy; simone.battista@edu.unige.it

**Keywords:** Scientific English, curriculum development, higher education, physical therapy modalities, physical therapy specialty, education, public health professional, evidence-based medicine, evidence-based practice

## Abstract

The knowledge of the English language is fundamental for the application of evidence-based practice. Hence, this study explores, through an online survey, the (1) perceived and (2) actual level of Scientific English among Italian undergraduate (UGs) and postgraduate (PGs) physiotherapists. As for (1), the participants expressed their agreement with 10 statements regarding the attitude towards Scientific English through a 1–4 Likert-type scale, with consensus set at ≥70%. As for (2), an ad-hoc 10-point questionnaire was developed through a Delphi procedure, with a pass score set at ≥60%. The survey was completed by 421 participants (UG: 47%; PG: 53%). As for (1), consensus was achieved in both groups in 4 out of 10 statements, specifically the ones addressing the capability to fully understand a scientific paper and physiotherapy-specific language in English. As for (2), the mean score reached by both groups was below 60%. The participants who had studied or were studying at a university in southern Italy presented 2.56 [1.54; 4.24] times higher odds to fail the test. New strategies to bridge the gap in the knowledge of Scientific English in Italy should be developed, through the creation of a unique syllabus tailored to the needs of future physiotherapists.

## 1. Introduction

Nowadays, English has become the undisputed lingua franca of science, used in the academic world [1]. As reported by Bennet, it is the language used in the most important international journals and the medium of higher-level instruction in universities across the world [1]. Having a lingua franca allows researchers to communicate worldwide with a common language and share information that is useful for scientific development [2]. Besides, scientific articles written in English have a higher number of citations compared to those published in other languages once the impact of journal, the year of publication, and the paper length have been statistically controlled [2]. 

In the last few years, thanks to the introduction of the evidence-based practice (EBP) paradigm in the clinical medical field, research has become more and more important for clinicians [3,4]. The EBP paradigm entails the use of a scientific methodology to organise and apply evidence to clinical decision-making processes, health policies, and healthcare decisions [3,4]. In order to provide medical treatments in line with the EBP paradigm, clinicians are required to balance their knowledge of the best evidence and their clinical expertise with the patients’ preferences and beliefs [4]. Furthermore, in order to be able to identify, evaluate, and apply the best current evidence, a certain body of knowledge and expertise is required, including being able to read and critically evaluate scientific papers written in the English language [5]. Therefore, not only is the knowledge of Scientific English fundamental for those who work in research, but it is also fundamental for those who work in the clinical field.

As far as physiotherapists are concerned, some studies conducted on a sample of American, Australian, and Swedish physiotherapists showed a general positive orientation towards research [6,7,8]. In Italy, a recent study by Castellini et al. reported similar results, showing that more than 80% of the participants who took part in their study perceived EBP as useful and necessary for their clinical practice, and stated that they were familiar with the principles of EBP [9]. However, the majority of them showed a gap between their perceived and actual knowledge of EBP. This is supported by research showing that Mediterranean countries appear to have higher educational needs when compared to their northern European counterparts [10].

Numerous obstacles can inhibit the application of EBP: these may include patients’ preferences, lack of time, availability of resources, discrepancies between different guidelines, and the knowledge of the English language [11,12]. Therefore, clinicians may encounter difficulties in identifying and reading high-quality research if they are not able to access resources in their native language [13]. For instance, in the Physiotherapy Evidence Database (PEDro), which is one of the most used physiotherapy research databases for evidence, approximately 90% of the content is published in English [14].

Given these premises, it is clear that the knowledge of the English language is fundamental for continuing education in medicine and for the professional updating of the individual. Identifying whether or not there are gaps in the knowledge of the English language becomes essential, as it can be the first step to sensitising its importance among a population of health professionals (i.e., physiotherapists) whose treatments should comply with the EBP paradigm. Hence, this study aimed at identifying the attitude towards and level of knowledge of Scientific English in a cohort of Italian last-year Physiotherapy undergraduate students (UGs) and postgraduate physiotherapists (PGs), who had completed or were close to completing a postgraduate degree, to analyse differences between undergraduate and postgraduate educational levels in this field.

## 2. Materials and Methods

### 2.1. Study Design 

A quantitative web-based cross-sectional survey investigating the attitude towards and the level of knowledge of Scientific English of physiotherapists in Italy (both UGs and PGs) was developed according to the *International Handbook of Survey Methodology*, through the use of distinct and iterative steps [15,16]. The study was conducted following the Declaration of Helsinki. Ethical approval was obtained from the Ethics Committee for University Research (CERA: Comitato Etico per la Ricerca di Ateneo), University of Genova (approval date: 27/07/2020; CERA2020.14), and follows the Strengthening the Reporting of Observational Studies in Epidemiology (STROBE) recommendations for reporting observational studies and the Checklist for Reporting Results of Internet E-Survey [17].

### 2.2. Survey Development

The questionnaire was developed by an expert in Scientific English (R.C.), an Italian with native-speaker-competence in English, who also has teaching experience in Higher Education (HE) in several degree courses for the Health Professions, together with a specialised physiotherapist in rehabilitation of musculoskeletal disorders and PhD Student (S.B.). The online version of the questionnaire was delivered through Microsoft 365 Forms, a secure web application to build and manage online surveys and databases, respecting the European General Data Protection Regulations [18]. The questionnaire included a brief cover letter and the informed consent outlining the aim of the study. The cover letter specified that participation in the survey was voluntary and that anonymity and confidentiality were guaranteed. 

The questionnaire contained 29 questions and it was divided into three sections: (1) demographic characteristics, (2) attitude towards Scientific English, and (3) knowledge of Scientific English. The first two sections were delivered in Italian, while the third one was in English. In the first section (questions 1–9), the participants were asked to provide information about their sex, age, education level, years of professional experience as a physiotherapist, the geographical area of the university at which they obtained their BSc Degree in Physiotherapy, years of study of the English language, years lived in an English-speaking country, and any English language certification obtained in the last five years. In the second section (questions 10–19), ten statements regarding the attitudes towards Scientific English were shown (Table 1), and the participants were asked to choose to what extent they agreed or disagreed with each statement through a 4-point Likert type scale ranging from 1 (completely disagree) to 4 (completely agree) [19]. 

Finally, the third section analysed the real level of knowledge of Scientific English. Since no test was retrieved from the literature, an ad hoc questionnaire was developed through a Delphi procedure. In order to create the questionnaire, R.C. and S.B. retrieved four physiotherapy papers (P1, P2, P3, P4) published between 2016 and 2018 in peer-reviewed journals (P1: 2017, P2: 2017, P3: 2016, P4: 2018). They extracted a total of five excerpts (P1: E1, P2: E2, P3: E3–E4, P4: E5), averaging 60 words each (E1: 45, E2: 36, E3: 94, E4: 59, E5: 67). Each of the five excerpts presented two questions (q1–q10), for a total of 10 questions (E1: q1–q2, E2: q3–q4, E3: q5–q6, E4: q7–q8, E5: q9–q10) aimed at investigating the participants’ level of comprehension of written texts (q1, q4, q5, q7, q8, q9) and linguistic competence at the syntactic, logical, and lexical levels (q2, q3, q6, q10).

The questionnaire was then sent to a panel of five experts in Scientific English, all with experience in teaching Scientific English at either undergraduate or postgraduate level, for face and content validity. R.C. sent an email to the panel experts individually, asking them for feedback on the reliability of the test, and on how they would assess the score of each question. All experts were unaware of the other participants in the Delphi procedure. In case of doubts, the participants could only email R.C., who would then report the comments received by all the experts to each component individually. After three rounds, a consensus (>70%) was reached. The panel agreed that all questions would be scored equally (1 point (pt) per correct answer, 0 pts per incorrect answer), and set the pass-score with six correct answers out of ten (60%), and all the items were considered consistent with the aim of the questionnaire. Finally, before the online dissemination, the whole survey was tested on a sample of five physiotherapists specialised in the rehabilitation of musculoskeletal or neurological disorders, so as to test its face validity. All of the sample participants fed back that the questionnaire was in line with its proposed objectives.

### 2.3. Participants

Participants were considered eligible to partake in this study if they were either attending the last year of a BSc degree in Physiotherapy (UGs) or if they were physiotherapists who had completed or were close to completing a postgraduate degree (PGs) in Italy. Therefore, UGs who were not attending the last year of a BSc degree in Physiotherapy or physiotherapists who had not attended or were not attending a PG degree were excluded. The exclusion criterion applied for UGs was adopted to make sure that all participants had already concluded all the credits attributed to the Scientific English course foreseen in their curriculum. Specifically, after the cover letter, the questionnaire included a preliminary question asking the respondents if they were attending the last year of the BSc in Physiotherapy or if they were physiotherapists working in Italy. Participants who answered “No” were shown a Thank You page and were not allowed to continue the questionnaire. The online version of the questionnaire, attainable through a hyperlink, was delivered through the newsletters of different Italian universities.

### 2.4. Variables

The primary outcomes of the present study were the attitudes towards and the knowledge of Scientific English of a cohort of Italian UGs and PGs.

### 2.5. Analysis

#### 2.5.1. Section 1: Demographic Characteristics

Descriptive analysis was carried out to understand the sample’s characteristics. In particular, the continuous variable “years lived in an English-speaking country” was reported as median (Q1: first quartile, Q3: third quartile) since it did not follow normal distribution, as reported in the investigation of the kurtosis and skewness indexes of the probability density functions and the exploration of the Q-Q plots. The continuous variables “years of studying the English language” and “age” followed a normal distribution and were reported as mean ± standard deviation (SD). Categorical outcomes (i.e., sex, educational level, years of professional experience as a physiotherapist, the geographical area of the university at which they obtained their BSc degree in Physiotherapy, and English language certifications) are reported as frequencies and percentages. As far as between-group analyses are concerned, an unpaired t-test was used to determine the difference in the variable “years of studying the English language” between UGs and PGs. The 95% confidence interval (95% CI) was calculated to estimate the magnitude of the effect. The Mann–Whitney U test was used to determine the difference in the variable “years lived in an English-speaking country”, and the effect size was calculated as $r=\frac{Z}{\sqrt{N}}$. 

#### 2.5.2. Section 2: Attitude towards Scientific English

In section two, the overall consensus with each statement was investigated for both UGs and PGs. The participants who partially or completely agreed (scores 3–4) were considered to agree with the statements. In the absence of a standard threshold, we defined a ≥70% agreement with a statement as consensus [20,21]. The frequencies of answers were calculated and a visual representation through a bar chart graph is reported. 

#### 2.5.3. Section 3: Knowledge of Scientific English

The frequencies of students and physiotherapists who reached a score ≥ 60% (pass score) were calculated and reported in percentages. The chi-square test was applied to compare the frequency of people who reached the above-mentioned pass score between the two groups. Moreover, the odds ratio (OR) of not passing (<60%) the test in UGs versus PGs was reported together with its 95% CI.

One logistic regression with a Wald backward method was performed to ascertain the effects of sex, age, education level, the geographical area of the university at which the participants obtained their BSc degree in Physiotherapy, years spent studying the English language, and English language certifications attained in the last five years on the likelihood that participants would reach the pass score in the test. The linearity of the continuous variables for the logit of the dependent variable was assessed via the Box-Tidwell procedure. A Bonferroni correction was applied using all twelve terms in the model, resulting in statistical significance being accepted when *p* < 0.01. Based on this assessment, all continuous independent variables were found to be linearly related to the logit of the dependent variable. There was no standardised residual assessing in the case-wise list. Odds ratio (OR) and 95% CI were estimated for each covariate reference category.

### 2.6. Sample Size Calculation

The sample size calculation formula reported by Taherdoost et al. for the calculation of the sample size in online surveys was used [22]. Specifically, the sample size was the number of completed responses expected to be received. Based on the numbers of Italian physiotherapists enrolled in the Italian professional register and Italian physiotherapist students attending the third year of the BSc degree in Physiotherapy, following the formula, setting a 5% margin of error (how accurately the results of the survey would reflect the views of the general population) and a sampling confidence level of 95% (how confident we could be that the population would select an answer within a certain range), the calculated sample size necessary for this study was 370.

## 3. Results

### 3.1. Section 1: Demographic Characteristics

Through the mailing lists of the Italian universities which agreed to disseminating the survey, 424 participants were reached between 11 November 2020 and 1 February 2021, among whom 3 did not provide consent to participate in the study and were thus excluded. Therefore, 421 participants (mean age 25.77 ± 5.46; female 49%, male 51%) were included in the analysis (Table 2). Among our cohort, 199 participants (mean age 23.06 ± 3.89; female 57%, male 43%) were UGs and 222 (mean age 28.20 ± 5.60; female 40%, male 60%) were PGs. 

Table 3 reports the expertise in the English language acquired by the participants expressed through (a) attained certifications in English as a Foreign Language in the last five years, (b) years spent studying English, and (c) years spent in a country where they used English as the vehicular language.

### 3.2. Section 2: Attitude towards Scientific English

**Figure 1:**
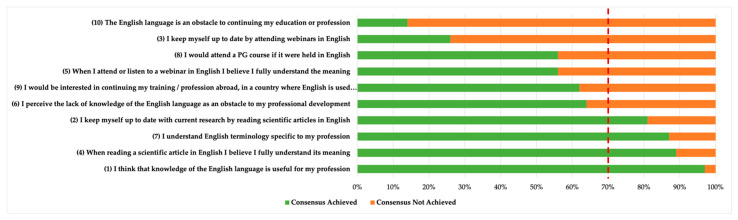
Level of consensus among UGs and PGs. Note: the red line represents the 70% level of consensus.

Overall, consensus was achieved for the same four (40%) statements (1, 2, 4, and 7) out of ten (Figure 1) by both UGs and PGs. These statements addressed the importance of Scientific English for the profession, the capability to fully understand a scientific paper written in English, the ability to understand the technical language of the profession in English, and of the habits of keeping themselves up to date by reading scientific articles in English.

### 3.3. Section 3: Knowledge of Scientific English

Among our sample, the mean score achieved by both UGs and PGs did not reach 60%, which had been set as the pass score (Table 4). However, the OR of achieving a score under 60% in the test for UGs versus PGs was 1.72 (CI 95%: [1.18; 12.6]) (Table 4). 

Table 5 categorises the ten questions into two groups: (a) comprehension (q1, q4, q5, q7, q8, q9) and (b) competence (q2, q3, q6, q10), and shows the number of respondents who answered each question correctly. 

Moreover, the binomial logistic regression was performed to ascertain the effects of sex, age, education level, the geographical area of the university at which the participants obtained their BSc degree in Physiotherapy, years spent studying the English language, and English language certifications attained in the last five years on the likelihood that participants would reach the pass score in the test. The logistic regression model was statistically significant, χ^2^(4) = 13.96, *p* < 0.001. Among the above-mentioned predictor variables, only the variable “geographical area of the university at which participants obtained their BSc degree in Physiotherapy” was significant. Participants who had studied or were studying at a university in southern Italy presented 2.56 (CI 95% [1.54; 4.24]) times higher odds to fail the test. 

## 4. Discussions

Our data provide insights on the actual and perceived knowledge of and attitudes towards Scientific English in PG Italian physiotherapists and final-year (UG) Physiotherapy students in the following areas, specifically: (A) insufficient levels of understanding of scientific articles written in English, (B) gap between actual and self-assessed level of understanding, (C) previous educational deficit, and (D) diatopic/geographical educational gap at national level. 

Regarding (A) and (B), when comparing Table 5—reporting the scores for the two macro-categories in which the questions have been grouped, comprehension and competence—with the answers reported in Figure 1 regarding the perceived competence of the Scientific English language, two observations may be brought to the forefront: one on the participants’ linguistic abilities, and the other on the gap between the actual and the perceived level of understanding of written English. The former highlights that the participants were better able to answer the lexico-syntactic competence questions correctly, which can be done regardless of their understanding of the article. However, none of the “comprehension” questions were answered correctly by 60% of the participants, which indicates that the level of understanding of an article in English by the population at stake is not sufficient. This could entail major repercussions on the application of EBP in physiotherapy practices, as English is arguably the lingua franca of academia, with more than 90% of publications in science written in this language [2].

However, a study conducted by Castellini et al. on 1289 Italian physiotherapists showed that when asked to rank ten possible barriers to EBP from 1st to 10th place (1st being the greatest barrier and 10th being the smallest), 696 participants (54,8%) ranked the “Language of scientific publications” at 8th, 9th, or 10th place, thus indicating that Italian physiotherapists do not perceive their proficiency in English, or rather their lack thereof, as a barrier against keeping up to date with published research and implementing it in everyday practice [9]. This evidence, combined with the perceived confidence in Scientific English reported by our cohort in Figure 1, allows our discussion to move towards the latter, and to highlight a noticeable gap between the participants’ self-assessment of their proficiency in English, and the results that the survey brought forth. More than 85% of the participants agreed that they can fully understand a scientific article in English, especially if containing specific terminology related to physiotherapy; however, the comprehension questions in our survey were answered correctly by less than 60% of the participants. Studies have shown that this is not uncommon, as the self-assessment process can present flaws since people have a tendency to overrate themselves as above average [23,24,25,26], and to inaccurately identify areas of weakness [27]. Moreover, it has been shown that people are inclined to overestimate their performance and misevaluate the skills they believe they have acquired [28,29], a phenomenon known as the Dunning–Kruger effect [30]. In light of this, it is possible to assert that the Italian UGs’ and PGs’ overrated self-assessment of their English language proficiency may result in their unwillingness to upskill in this area, which would subsequently hinder the application of EBP in their clinical setting, resulting in misapplication or non-application of the evidence retrieved from articles written in English.

As far as (C) is concerned, our results highlighted that, although UGs achieved a higher odds ratio of not passing this English test, among our sample, the mean score reached by both UGs and PGs did not yield 60%. The fact that the two investigated populations did not achieve the set pass-level mean score highlighted that an educational deficit is to be looked for in previous levels of education. In a web-based survey study on Italian English school teachers and university professors, Faez showed that almost all participants agreed or strongly agreed that English language teaching methods in Italy need to be improved or further developed [31]. Furthermore, as reported in EF’s 2020 EPI (English Proficiency Index) report, Italy only achieved a moderate level of proficiency in English on a five-point scale from Very High to Very Low, where Moderate was the mid-level [32]. Hence, it is possible to hypothesise that undergraduate or postgraduate courses which require students to pass a bespoke English language exam, but at the same time only allocate it a limited amount of CFU (Italian ECTS) in their curriculum, such as the ones for healthcare professionals in Italy for example, may not be able to overcome this educational gap. 

As regards (D), it is interesting to notice that, from a geographical point of view, physiotherapists who were attending or had obtained their BSc degree from southern universities showed a higher odds ratio of not attaining the pass score in the questionnaire. This is in line with Abramo et al.’s findings, which showed a discrepancy between the productivity of the educational system between northern and southern Italy [33]. As they asserted, the reason behind the north–south gap could stem from different origins, grounded in different economic, social, cultural, and historical-geographical roots. Moreover, the above-mentioned EF’s 2020 EPI report highlighted that within the Eurozone, Italy lags behind other northern member states, as far as English proficiency is concerned [32]. 

Some limitations of this study need to be discussed. Firstly, the cross-sectional nature of the study did not allow for an evaluation of the causative relationship between the sample’s demographic and educational characteristics and the knowledge of the English language. Secondly, we did not investigate the PG physiotherapists’ clinical practice setting (e.g., private practice, public care, etc.), which might have had an impact on the participants’ level of English knowledge. Thirdly, we did not ask those participants who admitted having attended other HE courses besides their degree in which course this was and where they had attended it. In addition, we did not have the possibility to calculate the response rate of this survey and, therefore, response bias may not be excluded. Finally, we did not investigate the participants’ competence in receptive oral skills (listening) and in productive skills (written or spoken). Although these competences are fundamental to allow clinicians to be more inclusive with patients from different nationalities, this was beyond the objectives of this study, which was more focussed on the application of the EBP paradigm. Future studies should investigate the above-mentioned competences to trace a broader picture of Italian physiotherapists’ proficiency in the English language. 

To conclude, we believe that our results should be considered at a policy and educational level to provide policymakers with evidence about the level of knowledge of Scientific English of Italian UGs and PGs, so that future decisions regarding HE courses in the health professions can be made accordingly. First and foremost, it is fundamental to stress the importance of developing critical thinking for self-assessment abilities among physiotherapists, in light of their lack of awareness about their real level of knowledge of Scientific English. Secondly, it is important to find new strategies to bridge the gap in the knowledge of Scientific English in HE, such as by reducing the heterogeneity of English courses among the different Italian universities by creating a unique and specialised syllabus tailored to the needs of future physiotherapists. 

## Figures and Tables

**Table 1:** Statements about the attitudes towards Scientific English.

Statements
(1) I think that the knowledge of the English language is useful for my profession.
(2) I keep myself up to date with current research by reading scientific articles in English.
(3) I keep myself up to date by attending webinars in English.
(4) When reading a scientific article in English, I believe I fully understand its meaning.
(5) When I attend or listen to a webinar in English, I believe I fully understand the meaning.
(6) I perceive the lack of knowledge of the English language as an obstacle to my professional development.
(7) I understand English terminology specific to my profession.
(8) I would attend a PG course if it were held in English.
(9) I would be interested in continuing my training/profession abroad, in a country where English is used as a vehicular language.
(10) The English language is an obstacle to continuing my education or profession.

**Table 2:** Participants’ demographic characteristics.

Demographic Data	Total	UGs	PGs
	(*n* = 421)	(*n* = 199)	(*n* = 222)
Age (years)(mean (SD))	25.77 (5.46)	23.06 (3.80)	28.20 (5.60)
Sex (female); (male) (*N* (%))	204 (49); 217 (51)	114 (57); 85 (43)	90 (40); 132 (60)
BSc University—Geographical Area (N (%))			
North	260 (62)	107 (62)	153 (69)
Centre	73 (17)	22 (17)	51 (23)
South	88 (21)	70 (21)	18 (8)
HE Level attained or in course (*N* (%))			
Third-Year Student BSc in Physiotherapy	199 (47)	199 (100)	/
I Level Postgraduate Certificate *	205 (49)	/	205 (92)
Master of Science (MSc)	17 (4)	/	17 (8)
Years of Work (*n* (%)) ^†^			
From 1 to 5 years	/	/	129 (31)
From 6 to 10 years	/	/	69 (16)
More than 10 years	/	/	24 (6)

Legend: UG, undergraduates; PG, postgraduates; N, number; SD, standard deviation; %, percentage; HE, higher education. * Academic degree that can be gained after BSc (Italian education system); ^†^ *n* = 222 (Physiotherapists with a degree).

**Table 3:** Acquired expertise in English.

Familiarity with the English Language	Total	UGs	PGs	
	(*n* = 421)	(*n* = 199)	(*n* = 222)	
English Certifications or CEFR level attained in the last five years (N (%)):				
No	300 (71)	199 (60)	181 (82)	
A1	3 (1)	3 (2)	0 (0)	
A2	6 (1)	4 (2)	2 (1)	
B1	39 (9)	25 (12)	14 (6)	
B2	61 (14)	40 (20)	21 (10)	
C1	11 (3)	7 (3)	4 (1)	
C2	1 (1)	1 (1)	0 (0)	
				Between Group
				Differences
				CI 95%
Years spent studying English (mean, (SD))	10.14 (3.81)	10.57 (3.81)	9.77 (3.80)	0.80 [0.07; 1.53] *
				Between Group
				Differences
				Effect Size
Years lived in an English Country (median (Q1; Q3))	0.0 (0; 0)	0.0 (0; 0)	0.0 (0; 0)	–0.09

Legend: UGs, undergraduates; PGs, postgraduates; *N*, number; CEFR, Common European Framework of Reference for Languages; %, percentage; Q1, first quartile; Q3, third quartile, * *p*-value < 0.01.

**Table 4:** Participants’ total score in the Scientific English questionnaire.

Questionnaire Score	UGs	PGs	Odds Ratio (No/Yes)
	(*n* = 199)	(*n* = 222)	CI 95%
Score (mean (SD))	5.04 (2.01)	5.67 (2.09)	/
Pass (N (%))			1.72 [1.17; 2.53] *
No	112 (56)	95 (43)	
Yes	87 (44)	127 (57)	

Legend: UG, undergraduates; PG, postgraduates; N, number; %, percentage; * *p* < 0.01.

**Table 5:** Participants’ score in each question of the Scientific English questionnaire.

**Comprehension**											
**q1 (N, %)**		**q4 (N, %)**		**q5 (N, %)**		**q7 (N, %)**		**q8 (N, %)**		**q9 (N, %)**	
**C**	**NC**	**C**	**NC**	**C**	**NC**	**C**	**NC**	**C**	**NC**	**C**	**NC**
226 (54)	195 (46)	229 (54)	192 (46)	221 (53)	201 (47)	83 (20)	338 (80)	242 (58)	234 (56)	234 (56)	187 (44)
**Competence**											
**q2 (N, %)**		**q3 (N, %)**		**q6 (N, %)**		**q10 (N, %)**					
**C**	**NC**	**C**	**NC**	**C**	**NC**	**C**	**NC**				
260 (62)	161 (38)	368 (87)	53 (13)	221 (53)	121 (29)	279 (66)	142 (34)				

Legend: q, question; N, number; %, percentage; C, correct; NC, not correct.

## Data Availability

The datasets used and analysed during the current study are available from the corresponding author upon reasonable request.

## References

[1] Bennett K. (2013). English as a lingua franca in academia: Combating Epistemicide through Translator Training. Interpret. Transl. Train..

[2] Di Bitetti M.S., Ferreras J.A. (2017). Publish (in English) or perish: The effect on citation rate of using languages other than English in scientific publications. Ambio.

[3] Lewis S.J., Orland B.I. (2004). The importance and impact of evidence-based medicine. J. Manag. Care Pharm..

[4] Sackett D.L., Rosenberg W.M.C., Gray J.A.M., Haynes R.B., Richardson W.S. (2007). Evidence based medicine: What it is and what it isn’t. 1996. Clin. Orthop. Relat. Res..

[5] Shaughnessy A.F., Torro J.R., Frame K.A., Bakshi M. (2016). Evidence-based medicine and life-long learning competency requirements in new residency teaching standards. Evid. Based. Med..

[6] Jette D.U., Bacon K., Batty C., Carlson M., Ferland A., Hemingway R.D., Hill J.C., Ogilvie L., Volk D. (2003). Evidence-Based Practice: Beliefs, Attitudes, Knowledge, and Behaviors of Physical Therapists. Phys. Ther..

[7] Iles R., Davidson M. (2006). Evidence based practice: A survey of physiotherapists’ current practice. Physiother. Res. Int..

[8] Kamwendo K. (2002). What do Swedish physiotherapists feel about research? A survey of perceptions, attitudes, intentions and engagement. Physiother. Res. Int..

[9] Castellini G., Corbetta D., Cecchetto S., Gianola S. (2020). Twenty-five years after the introduction of Evidence-based Medicine: Knowledge, use, attitudes and barriers among physiotherapists in Italy—A cross-sectional study. BMJ Open.

[10] Vliet Vlieland T.P.M., Van Den Ende C.H.M., Alliot-Launois F., Beauvais C., Gobbo M., Iagnocco A., Lundberg I.E., Munuera-Martínez P.V., Opava C.H., Prior Y. (2016). Educational needs of health professionals working in rheumatology in Europe. RMD Open.

[11] Amano T., González-Varo J.P., Sutherland W.J. (2016). Languages Are Still a Major Barrier to Global Science. PLoS Biol..

[12] Fischer F., Lange K., Klose K., Greiner W., Kraemer A. (2016). Barriers and Strategies in Guideline Implementation—A Scoping Review. Healthcare.

[13] Sadeghi-Bazargani H., Tabrizi J.S., Azami-Aghdash S. (2014). Barriers to evidence-based medicine: A systematic review. J. Eval. Clin. Pract..

[14] Yamato T.P., Arora M., Stevens M.L., Elkins M.R., Moseley A.M. (2018). Quality, language, subdiscipline and promotion were associated with article accesses on Physiotherapy Evidence Database (PEDro). Physiotherapy.

[15] de Leeuw D., Hox J.D.D. (2008). International Handbook of Survey Methodology.

[16] Eysenbach G. (2004). Improving the Quality of Web Surveys: The Checklist for Reporting Results of Internet E-Surveys (CHERRIES). J. Med. Internet Res..

[17] Von Elm E., Altman D., Egger M., Pocock S., Gøtzsche P., Vandenbroucke J. (2007). Strobe Initiative The Strengthening the Reporting of Observational Studies in Epidemiology (STROBE) statement: Guidelines for reporting observational studies. Ann. Intern. Med..

[18] (2016). Regulation (EU) 2016/679 of the European Parliament and of the Council of 27 April 2016 on the Protection of Natural Persons with Regard to the Processing of Personal Data and on the Free Movement of Such Data, and Repealing Directive 95/46/EC. https://op.europa.eu/en/publication-detail/-/publication/3e485e15-11bd-11e6-ba9a-01aa75ed71a1/language-en.

[19] Cooper I.D., Johnson T.P. (2016). How to use survey results. J. Med. Libr. Assoc..

[20] Teo P.L., Hinman R.S., Egerton T., Dziedzic K.S., Bennell K.L. (2019). Identifying and Prioritizing Clinical Guideline Recommendations Most Relevant to Physical Therapy Practice for Hip and/or Knee Osteoarthritis. J. Orthop. Sport. Phys. Ther..

[21] Battista S., Salvioli S., Millotti S., Testa M., Dell’Isola A. (2021). Italian physiotherapists’ knowledge of and adherence to osteoarthritis clinical practice guidelines: A cross-sectional study. BMC Musculoskelet. Disord..

[22] Taherdoost H. (2017). Determining Sample Size; How to Calculate Survey Sample Size. Int. J. Econ. Manag. Syst..

[23] Pisklakov S., Rimal J., McGuirt S. (2014). Role of Self-Evaluation and Self-Assessment in Medical Student and Resident Education. Br. J. Educ. Soc. Behav. Sci..

[24] Dunning D., Heath C., Suls J.M. (2004). Flawed self-assessment implications for health, education, and the workplace. Psychol. Sci. Public Interes. Suppl..

[25] Davis D.A., Mazmanian P.E., Fordis M., Van Harrison R., Thorpe K.E., Perrier L. (2006). Accuracy of physician self-assessment compared with observed measures of competence: A systematic review. J. Am. Med. Assoc..

[26] Mort J.R., Hansen D.J. (2010). First-year pharmacy students’ self-assessment of communication skills and the impact of video review. Am. J. Pharm. Educ..

[27] Regehr G., Eva K. (2006). Self-assessment, self-direction, and the self-regulating professional. Clin. Orthop. Relat. Res..

[28] Baxter P., Norman G. (2011). Self-assessment or self deception? A lack of association between nursing students’ self-assessment and performance. J. Adv. Nurs..

[29] Galbraith R., Hawkins R., Holmboe E. (2008). Making self-assessment more effective. J. Contin. Educ. Health Prof..

[30] Kruger J., Dunning D. (2000). Unskilled and Unaware of It: How Difficulties in Recognizing One’s Own Incompetence Lead to Inflated Self-Assessments. J. Pers. Soc. Psychol..

[31] Faez F. (2011). English Education in Italy: Perceptions of Teachers and Professors of English. Comp. Int. Educ..

[32] EF English Proficiency Index. https://www.ef.com/assetscdn/WIBIwq6RdJvcD9bc8RMd/legacy/__/~/media/centralefcom/epi/downloads/full-reports/v10/ef-epi-2020-english.pdf.

[33] Abramo G., Angelo C.A., Rosati F. (2016). The north-south divide in the Italian higher education system. Scientometrics.

